# CO_2_-Selective Nanoporous Metal-Organic Framework Microcantilevers

**DOI:** 10.1038/srep10674

**Published:** 2015-06-02

**Authors:** Changyong Yim, Moonchan Lee, Minhyuk Yun, Gook-Hee Kim, Kyong Tae Kim, Sangmin Jeon

**Affiliations:** 1 Department of Chemical Engineering, Pohang University of Science and Technology (POSTECH), Pohang, Gyeongbuk, Republic of Korea; 2 Clean Coal Chemicals Research Project, Research Institute of Industrial Science and Technology (RIST), Pohang, Gyeongbuk, Republic of Korea

## Abstract

Nanoporous anodic aluminum oxide (AAO) microcantilevers are fabricated and MIL-53 (Al) metal-organic framework (MOF) layers are directly synthesized on each cantilever surface by using the aluminum oxide as the metal ion source. Exposure of the MIL53-AAO cantilevers to various concentrations of CO_2_, N_2_, CO, and Ar induces changes in their deflections and resonance frequencies. The results of the resonance frequency measurements for the different adsorbed gas molecules are almost identical when the frequency changes are normalized by the molecular weights of the gases. In contrast, the deflection measurements show that only CO_2_ adsorption induces substantial bending of the MIL53-AAO cantilevers. This selective deflection of the cantilevers is attributed to the strong interactions between CO_2_ and the hydroxyl groups in MIL-53, which induce structural changes in the MIL-53 layers. Simultaneous measurements of the resonance frequency and the deflection are performed to show that the diffusion of CO_2_ into the nanoporous MIL-53 layers occurs very rapidly, whereas the binding of CO_2_ to hydroxyl groups occurs relatively slowly, which indicates that the adsorption of CO_2_ onto the MIL-53 layers and the desorption of CO_2_ from the MIL-53 layers are reaction limited.

Metal-organic frameworks (MOFs) are organic-inorganic hybrid crystalline materials that consist of metal ions coordinated to organic linkers. They have attracted much attention as novel materials for catalysts,^1^ gas separation^2^, and gas storage^3^,^4^ because of their high surface areas, chemical versatility, and tunable pore sizes. Flexible crystalline MOFs can undergo significant structural changes during the adsorption or desorption of gas molecules; some MOFs increase in volume by more than 30% upon gas adsorption^5^. Such structural changes upon gas adsorption as well as their high surface areas and chemical versatility suggest that MOFs could be useful as sensing materials in next-generation gas sensors. However, the applications of MOFs in gas sensors have been limited because conventional sensors measure the changes in electrical properties upon gas adsorption but most MOFs are not conductive^6^.

This problem may be circumvented by using nanomechanical sensors as platforms for MOFs. Hesketh and Kitagawa synthesized MOFs on the microcantilever sensors and QCMs, and measured the effects of gas adsorption on their stress and mass respectively during the adsorption or desorption of various gases^7^,^8^,^9^,^10^. The stress measurements were found to be very sensitive to gas adsorption. However, the direct growth of MOFs on microcantilever surfaces produces the interface stress and unwanted bending of the cantilever degrades sensor performance. Several methods such as layer-by-layer coating^7^,^11^,^12^, dielectrophoresis^13^, and drop-casting^14^, have been developed for the synthesis of MOFs, but achieving the uniform and stress-free growth of MOFs on thin and flexible cantilevers remains a challenge.

In this study, we fabricated nanoporous anodic aluminum oxide (AAO) microcantilevers and synthesized MIL-53 (AI) MOF layers directly on the surfaces of the AAO microcantilevers by using the aluminum oxide as the metal ion source^15^,^16^,^17^. Interface stress due to the growth of MIL-53 is suppressed by the presence of the free space in the nanopores of the AAO cantilever. The MIL53-AAO cantilevers were exposed to N_2_, CO, Ar, and CO_2_ and the resulting changes in their resonance frequencies and deflections were measured simultaneously. The high surface areas and low Young’s moduli of these nanoporous cantilevers mean that they can be used to measure both of these changes sensitively. The cantilevers’ resonance frequencies were found to be insensitive to the type of adsorbed gas molecule but their deflections change only in response to the adsorption of CO_2_. To the best of our knowledge, this is the first report of the fabrication of nanoporous microcantilevers with MOF layers and their gas adsorption characteristics.

## Results and Discussion

### Fabrication Process of MIL53-AAO Microcantilevers

**Figure 1:**
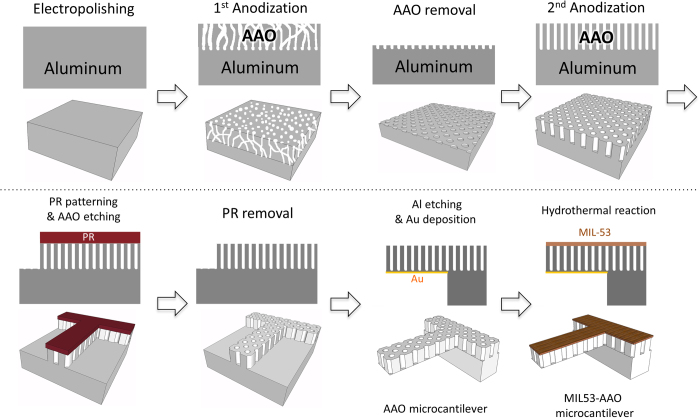
Schematic illustration of the fabrication of an AAO microcantilever and a MIL53-AAO microcantilever.

Figure 1 illustrates our process for the fabrication of the AAO microcantilevers (for more details, see the Experimental section)^18^,^19^,^20^. In brief, an electropolished aluminum sheet is anodized twice to produce hexagonally ordered nanopores. A thin layer of photoresist (PR) is spin-coated onto the sheet, then microstructures are patterned on the sheet by using UV irradiation. A thin Au layer is deposited on the back of the AAO microcantilever to prevent the growth of MIL-53 on that side of the sheet. A hydrothermal reaction is then performed to synthesize a MIL-53 (AI) layer directly on the AAO cantilever.

### Characterization of MIL53-AAO Microcantilevers

**Figure 2:**
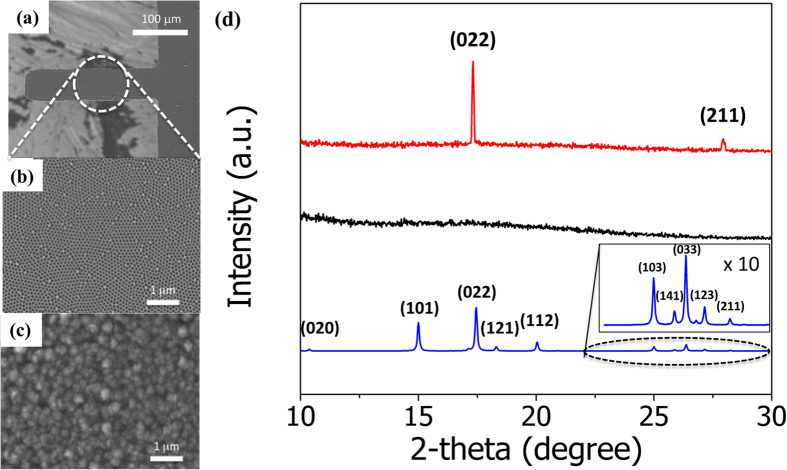
(**a**) SEM image of a MIL53-AAO microcantilever. Magnified images of the AAO microcantilever surface (**b**) before and (**c**) after synthesis of the MIL-53 layer. (**d**) X-ray diffraction patterns of the AAO substrate (black), the MIL-53 (AI) layer on the AAO substrate (red), and powder XRD patterns of MIL-53 (Al) from the reference (blue).

Figures 2a and b show scanning electron microscopy (SEM) images of an AAO microcantilever at different magnifications. The fabricated AAO microcantilever beam is 250 μm long, 50 μm wide, and 2 μm thick. The average pore diameter of the microcantilever is 50 nm and its pore-to-pore distance is ~100 nm. The hydrothermal reaction converts the aluminum oxide layer on the surface of the AAO cantilever to MIL-53 (AI). The MIL53-AAO microcantilevers were washed several times in water, then kept at 100 °C in an oven under reduced pressure to remove water molecules, which produced the MIL-53 (Al) with the open-pored structure. The SEM image of the MIL-53 layer in Fig. 2c shows that it consists of granules that are a few hundred nanometers in diameter. There are no peaks in the range 10° ≤ 2θ ≤ 30° in the XRD pattern for the AAO substrate (obtained from a separate sheet of MIL53-AAO), whereas that of the MIL-53 layer on the AAO substrate contains (022) and (211) peaks (Fig. 2d)^21^, which indicates that the MOF layer has a highly-oriented crystalline structure. The MIL-53 layer is 100 nm thick (see also Figure S1) and the AAO layer is 2 μm thick. The thickness of the AAO substrate is only slightly affected by the growth of the MIL-53 layer.

**Figure 3:**
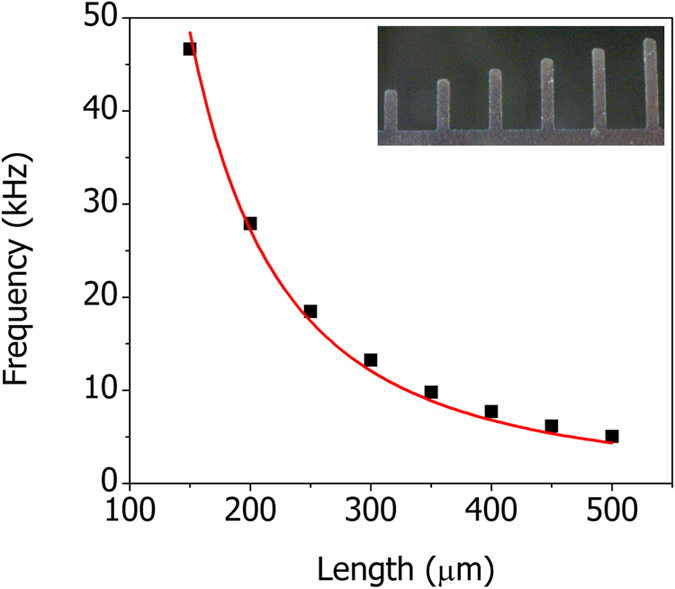
The resonance frequencies of the MIL53-AAO microcantilevers with various beam lengths. The inset shows an optical image of these cantilevers.

The inset in Fig. 3 shows an optical microscopy image of MIL53-AAO microcantilevers with various beam lengths. The microcantilever resonance frequency decrease as the beam length increases (Fig. 3); this decrease can be fitted with the composite beam equation (Equation 1):^22^
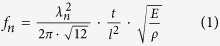
where *f*_*n*_, λ_*n*_, *t*, *l*, *E*, and ρ are the *n-*th resonant frequency, the *n-*th mode eigenvalue (λ_0_ = 1.875), the thickness, the length, the effective Young’s modulus, and the apparent density of the beam, respectively. Assuming that the moduli of the 100 nm thick MIL-53 layers and the 30 nm thick Au layers are negligible compared to the modulus of the 2 μm thick AAO cantilever, the calculated Young’s modulus of the MIL53-AAO cantilevers is 28.9 GPa, which is less than that of dense aluminum oxide (~140 GPa) or silicon (~170 GPa)^18^,^19^,^20^. The low moduli of the AAO cantilevers enable sensitive measurement of changes in the deflection that arise due to the gas adsorption-induced structural changes in MIL-53.

### Gas Sorption on AAO microcantilevers and MIL53-AAO microcantilevers

**Figure 4:**
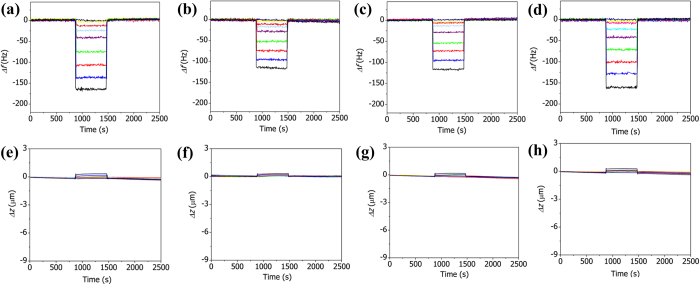
Variations in the resonance frequency (**a**-**d**) and deflection (**e**-**h**) of the AAO microcantilever during the adsorption and desorption of various concentrations of CO_2_ (**a**, **e**), N_2_ (**b**, **f**), CO (**c**, **g**), and Ar (**d**, **h**). The gas concentrations were increased over a series of measurements: 1% (dark blue) → 3% (yellow) → 5% (pink) → 10% (light blue) → 20% (purple) → 40% (green) → 60% (red) → 80% (blue) → 100% (black).

Figures 4a-d show the variations in the resonance frequency of the plain AAO microcantilever (250 μm long) during the adsorption and desorption of CO_2_, N_2_, CO, and Ar respectively. Helium was used as the carrier gas; the concentrations of the gas streams were varied from 0 to 100% by controlling the flow rates of helium and the analyte gas with mass flow controllers at a fixed total flow rate of 75 mL/min. The adsorption of CO_2_, N_2_, CO, or Ar molecules onto the cantilever induces instant decreases in its frequency. A decrease in the frequency of the AAO cantilever indicates that there has been an increase in the mass loading due to the adsorption of gas molecules. The largest change in the frequency was observed for the adsorption of CO_2_. However, the frequency changes produced by the adsorptions of the three gases are almost identical when normalized by the molecular weight of each gas (Figure S2a). This result indicates that the same number of gas molecules are adsorbed onto the cantilever at the same analyte gas concentrations and that no specific interactions occur between the AAO surface and CO_2_, N_2_, CO, or Ar molecules.

The variations in the deflection of the AAO microcantilever were measured simultaneously with changes in its resonance frequency, as shown in Fig. 4e-h. In contrast to the relatively large changes in the resonance frequency with the gas concentration, the change in the deflection is quite small; this observation indicates that the interaction between the AAO surface and the adsorbed gas molecules is so weak that the adsorption of gas molecules does not induce a large surface stress. The low selectivity of the frequency measurements and the low sensitivity of the deflection measurements imply that the AAO cantilever is not suitable for the investigation of gas adsorption.

**Figure 5:**
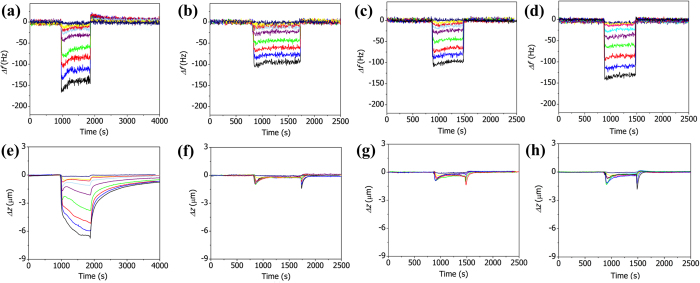
Variations in the resonance frequency (**a**-**d**) and deflection (**e**-**h**) of the MIL53-AAO microcantilever during the adsorption and desorption of various concentrations of CO_2_ (**a**, **e**), N_2_ (**b**, **f**), CO (**c**, **g**), and Ar (**d**, **h**). Gas concentrations were increased over a series of measurements: 1% (dark blue) → 3% (yellow) → 5% (pink) → 10% (light blue) → 20% (purple) → 40% (green) → 60% (red) → 80% (blue) → 100% (black).

Similar experiments were conducted using the MIL53-AAO microcantilevers under the same conditions as those used to determine the results in Fig. 4. Figures 5a-d show the variations in the resonance frequency of the MIL53-AAO microcantilevers during the adsorption and desorption of CO_2_, N_2_, CO, and Ar, respectively. The changes in the frequencies of the MIL53-AAO microcantilevers are similar to those of the AAO microcantilever, except that no overshooting was observed in the AAO microcantilever; this difference suggests that the MIL-53 layers are responsible for the overshoot, probably because gas adsorption induces structural change (i.e., modulus change) in the MIL-53 layers.

The gas adsorption-induced structural changes are more apparent in the deflection measurements than in the frequency measurements. Figures 5e-h show the variations in the deflection of the MIL53-AAO microcantilever during the adsorption and desorption of CO_2_, N_2_, CO, and Ar respectively. Although the number of adsorbed gas molecules does not vary with the type of gas at the same concentration (see Figure S2b for the normalized frequency change), only the adsorption of CO_2_ molecules induces significant changes in the deflection. The adsorptions of CO and N_2_ result in nearly negligible changes in the deflection, except during the early stages of adsorption and desorption. Note that the MIL53-AAO microcantilever bent negatively (i.e., bent away from its top layer (MIL-53 layer)) during gas adsorption whereas the AAO microcantilever bent positively (bent toward to its top layer). The deflection of the cantilever occurs due to the differential changes in the gas adsorption-induced surface stress between the top and bottom surfaces. The negative deflection of the MIL53-AAO cantilever implies that the effective surface area of MIL-53 is larger than that of AAO.

**Figure 6:**
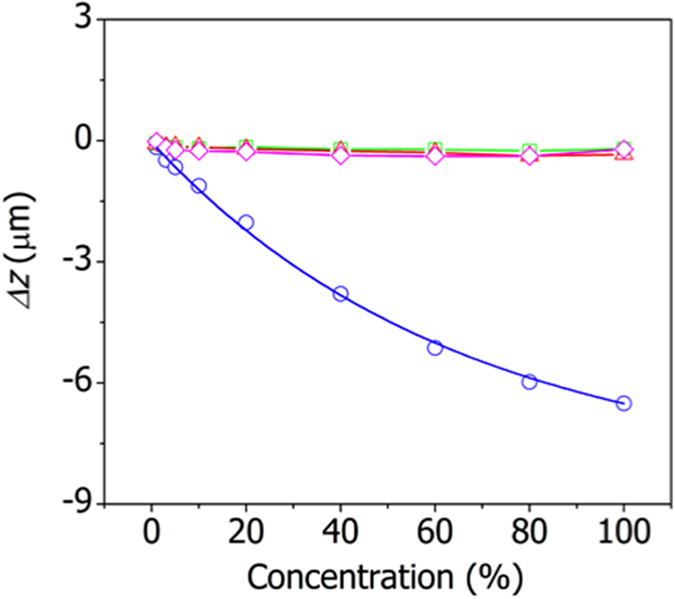
Variations in the deflection of the MIL53-AAO cantilever due to the adsorption of CO_2_ (blue circles), N_2_ (green squares), CO (red triangles), and Ar (magenta diamonds) at various concentrations. The solid lines are shown to guide the eye.

Figure 6 compares the changes in the deflection of the MIL53-AAO cantilever due to the adsorptions of CO_2_, N_2_, CO and Ar at various concentrations. Substantial changes in the deflection are evident only for the adsorption of CO_2_; nearly identical results were obtained when the experiments were repeated (Figure S3). The CO_2_ adsorption-induced deflection is ~30 times that due to the adsorption of CO, N_2_, or Ar. The high selectivity of the MIL53-AAO microcantilevers with respect to CO_2_ adsorption has been attributed to the strong interactions between CO_2_ and hydroxyl groups located at metal-oxygen-metal links in the MIL-53 framework^23^. The adsorption of CO_2_ onto the metal ion bridging hydroxyl groups in MIL-53 induces a change in the structure of the framework^24^. In addition, the cantilever’s low modulus means that the gas adsorption-induced structural changes are converted into large deflections. The high sensitivity of the microcantilever sensor made it possible to measure the structural changes of polymers during glass transition^25^,^26^,^27^,^28^.

**Figure 7:**
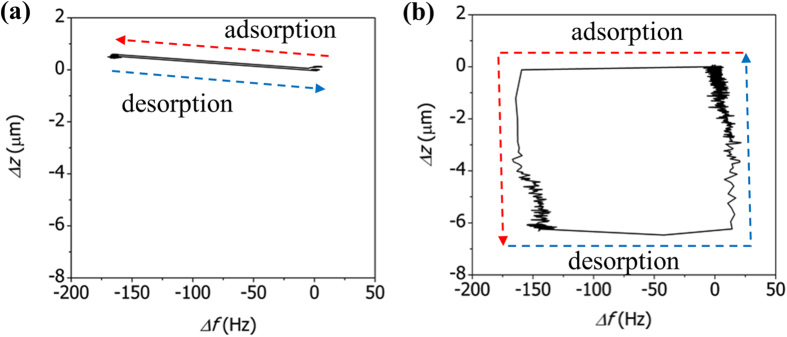
Variations in the deflection of (**a**) the AAO microcantilever and (**b**) the MIL53-AAO microcantilever with the change in the resonance frequency during the adsorption and desorption of CO_2_ at a concentration of 100%. (The red and blue arrows indicate the adsorption and desorption of CO_2_ respectively.).

Figure 7a shows the variation in the deflection of the AAO microcantilever with the changes in the resonance frequency during the adsorption and desorption of CO_2_ at a concentration of 100%. The adsorption of CO_2_ molecules onto the AAO cantilever induces simultaneous changes in the resonance frequency and the deflection of the cantilever. Hysteresis is negligible during the adsorption and desorption. In contrast, the adsorption of CO_2_ molecules onto the MIL53-AAO cantilever induces substantial hysteresis, as shown in Fig. 7b (see also Figure S4). During the early stages of CO_2_ adsorption, the resonance frequency decreases rapidly without changes in the deflection, then the cantilever bends downwards at a nearly constant resonance frequency. The frequency change is directly related to the mass loading due to CO_2_ adsorption, so this result indicates that the diffusion of CO_2_ into the nanoporous MIL-53 layer occurs very fast, whereas the binding of CO_2_ to hydroxyl groups in the MIL-53 framework occurs relatively slowly. This contrast explains why the structural changes in the MIL-53 layer (i.e., cantilever bending) continue at constant frequency. The slight increase in the resonance frequency during the final stage of CO_2_ adsorption might be due to a change in the structure (i.e., the increase in the modulus) of the MIL-53 layer. Similar responses are evident during the desorption of CO_2_ molecules. The resonance frequency increases rapidly without change in the deflection, then the cantilever bends upwards at a nearly constant resonance frequency.

## Conclusions

We synthesized MIL-53 (AI) MOF layers directly on nanoporous AAO microcantilevers by using the aluminum oxide layer on the surface of each cantilever as a metal ion source. The nanopores on the AAO cantilever surface were found to suppress the stress during the growth of the MIL-53 layer and to thus prevent excessive bending of the cantilever. The MIL53-AAO cantilever was exposed to CO_2_, N_2_, CO, and Ar, and found to exhibit selective and sensitive changes in deflection only for CO_2_. The selective response to CO_2_ is due to the strong interactions between CO_2_ and MIL-53, and its sensitivity is due to the low modulus of the nanoporous AAO cantilever. In particular, we performed simultaneous measurements of the changes in the frequency and deflection to demonstrate that the diffusion of CO_2_ into the nanoporous MIL-53 layer occurs very rapidly, whereas the binding of CO_2_ to hydroxyl groups in the MIL-53 layer occurs relatively slowly, which indicates that CO_2_ adsorption is reaction limited. Various MOF layers can easily be synthesized on nanoporous AAO cantilevers, so this approach has significant potential for the development of novel gas sensors and also for the characterization of MOFs.

## Methods

### Materials

Phosphoric acid, acetic acid, chromic acid, nitric acid, perchloric acid, oxalic acid, ethanol, acetone, and terephthalic acid were purchased from Sigma-Aldrich and used without further purification. The photoresist AZ 1512 and the AZ developer CD30 were obtained from Clariant (Somerville, NJ), and high purity aluminum sheet was obtained from Sigma-Aldrich. Deionized water (18.3 MΩ cm) was obtained by using a reverse osmosis water system (Human Science, Korea).

### Fabrication of the AAO microcantilever

AAO microcantilevers were fabricated as described previously^18^,^19^,^20^. An aluminum sheet was electropolished in a mixture of ethanol and perchloric acid (4:1 by vol.%) for 10 min, then anodized twice in 0.3 M oxalic acid to produce AAO with well-defined nanopores. The AAO substrate was cut into two pieces: one piece was used for the fabrication of an AAO microcantilever and the other was used for X-ray diffraction (XRD) analysis. To pattern microcantilevers on the AAO surface, a thin layer of the photoresist was spin-cast onto the aluminum sheet, then photolithography was performed. After the reacted photoresist was removed by rinsing in the AZ developer, the AAO sheet was wet-etched in a 5 wt.% phosphoric acid solution for 3 h. A suspended cantilever structure was obtained by performing electrochemical etching under electropolishing conditions for 1 h. A 3 nm Cr adhesion layer and a 30 nm Au layer were sequentially deposited onto the back of the AAO cantilever to prevent growth of MIL-53 on that side during the subsequent hydrothermal reaction. The Au layer also acts as a reflector; AAO is transparent, so does not reflect laser beams.

### Synthesis of MIL-53 (AI) on the AAO microcantilevers

MIL-53 (AI) is conventionally synthesized by using Al(NO_3_)_3_·9H_2_O and terephthalic acid as the metal ion source and the organic linker respectively. In this study, the MIL-53 layer was directly synthesized on each AAO microcantilever by using the aluminum oxide cantilever surface as the metal ion source. The AAO microcantilevers were immersed in a Teflon-lined autoclave containing 10 mM terephthalic acid and maintained at 200 °C for 1 h. The MIL53-AAO microcantilevers were washed several times in water, then kept at 100 °C in an oven under reduced pressure to remove water molecules, which produced the MIL-53 (Al) with the open-pored structure. The morphology and crystalline structure of the MOF layers were confirmed by using scanning electron microscopy (SEM) and X-ray diffraction (XRD) respectively.

### Instrument set-up

The fabricated microcantilevers were mounted on a gas flow cell equipped with an embedded piezo-actuator (Figure S5). A focused laser beam was reflected off the gold-coated cantilevers and their deflections were recorded with a position-sensitive detector (SiTek Electro Optics, Partille, Sweden). A fast Fourier transform (FFT) algorithm was used to convert the voltage changes due to the vibrations of the cantilevers to resonance peaks from which the resonance frequencies were calculated. All the experiments were conducted at room temperature.

## Additional Information

**How to cite this article**: Yim, C. *et al.* CO2-Selective Nanoporous Metal-Organic Framework Microcantilevers. *Sci. Rep.* **5**, 10674; doi: 10.1038/srep10674 (2015).

## Supplementary Material

Supporting Information
