# Supplementary material for: CO2-Selective Nanoporous Metal-Organic Framework Microcantilevers
Source: Sci Rep. 2015 Jun 2;5:10674. doi: 10.1038/srep10674 (PMC4451844; doi:10.1038/srep10674)
Supplement: Supporting Information [file srep10674-s1.doc]

**Supporting Information**

**CO_2_-Selective Nanoporous Metal-Organic Framework Microcantilevers**

Changyong Yim,^1^ Moonchan Lee,^1^ Minhyuk Yun,^1^ Gook-Hee Kim,^2^ Kyong Tae Kim ^2^ and Sangmin Jeon^1,*^

*^1^Department of Chemical Engineering, Pohang University of Science and Technology (POSTECH), Pohang, Gyeongbuk, Republic of Korea*

*^2^Clean Coal Chemicals Research Project, Research Institute of Industrial Science and Technology (RIST), Pohang, Gyeongbuk, Republic of Korea*

* Author to whom correspondence should be addressed. E-mail: jeons@postech.ac.kr


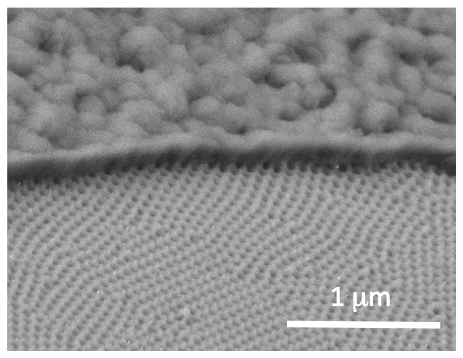


**Figure S1.** Tilted view of the SEM image of the MIL-53 AAO substrate. The thickness of MIL-53 layer was ~100 nm.


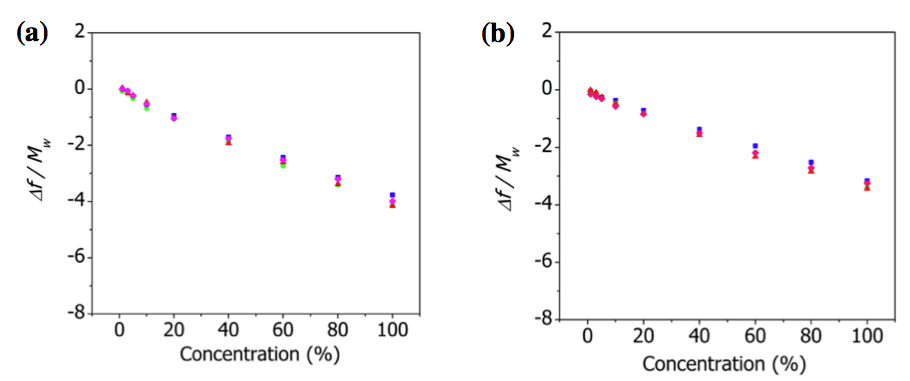


**Figure S2.** Changes in the normalized resonance frequency of (a) the AAO microcantilever and (b) the MIL53-AAO microcantilever with various concentrations of CO_2_ (blue squares), N_2_ (green circles), CO (red triangles), and Ar (magenta diamonds)

**
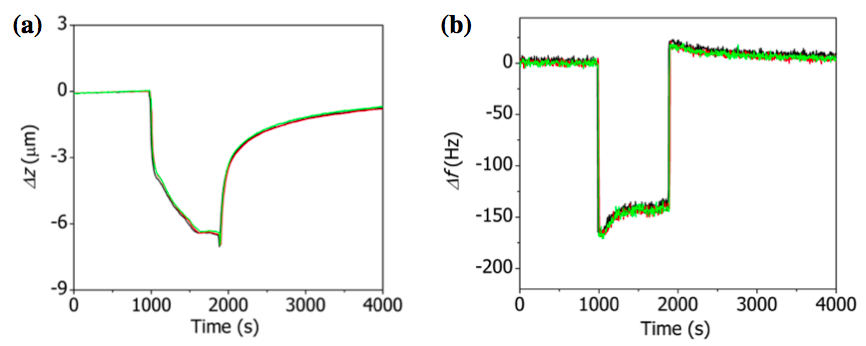
**

**Figure S3.** Changes in the (a) deflection and (b) resonance frequency of the MIL53-AAO microcantilever exposed to CO_2_ at a concentration of 100 % in three consecutive cycles.


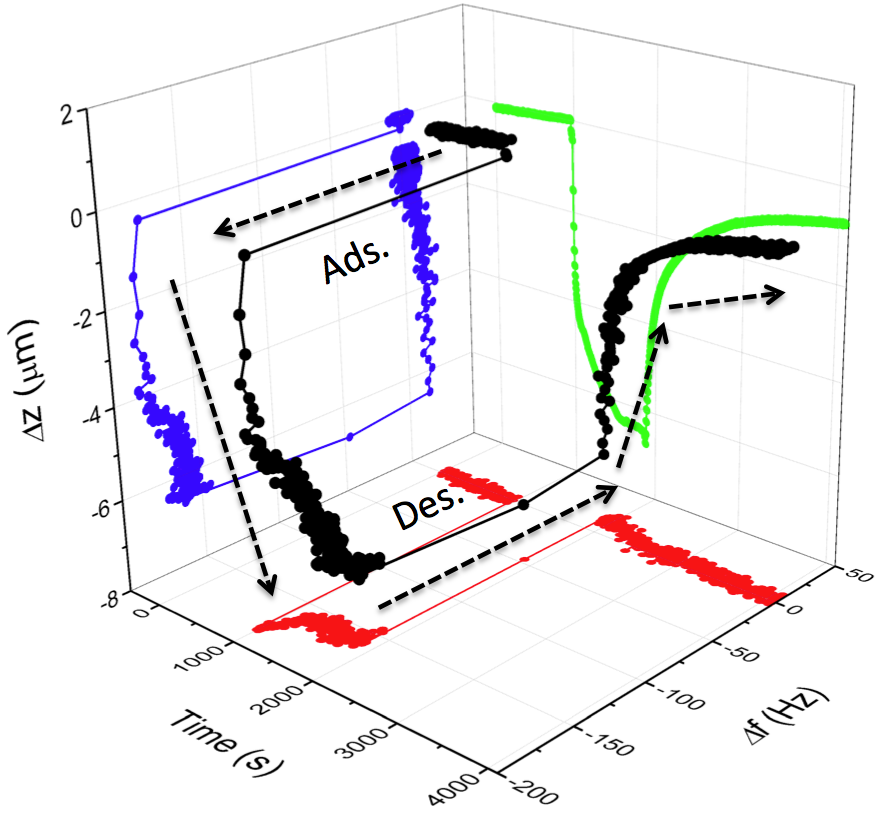


**Figure S4.** Changes in the deflection and resonance frequency of the MIL53-AAO microcantilever with time variations when exposed to CO_2_ at a concentration of 100 %; time-deflection (green), time-frequency (red), deflection-frequency (blue), and time-deflection-frequency (black). The projections of the time-deflection-frequency signals on each plane (red, green, blue) correspond to Figure 5(a), 5(e), and 7(b) of the main text, respectively.

**
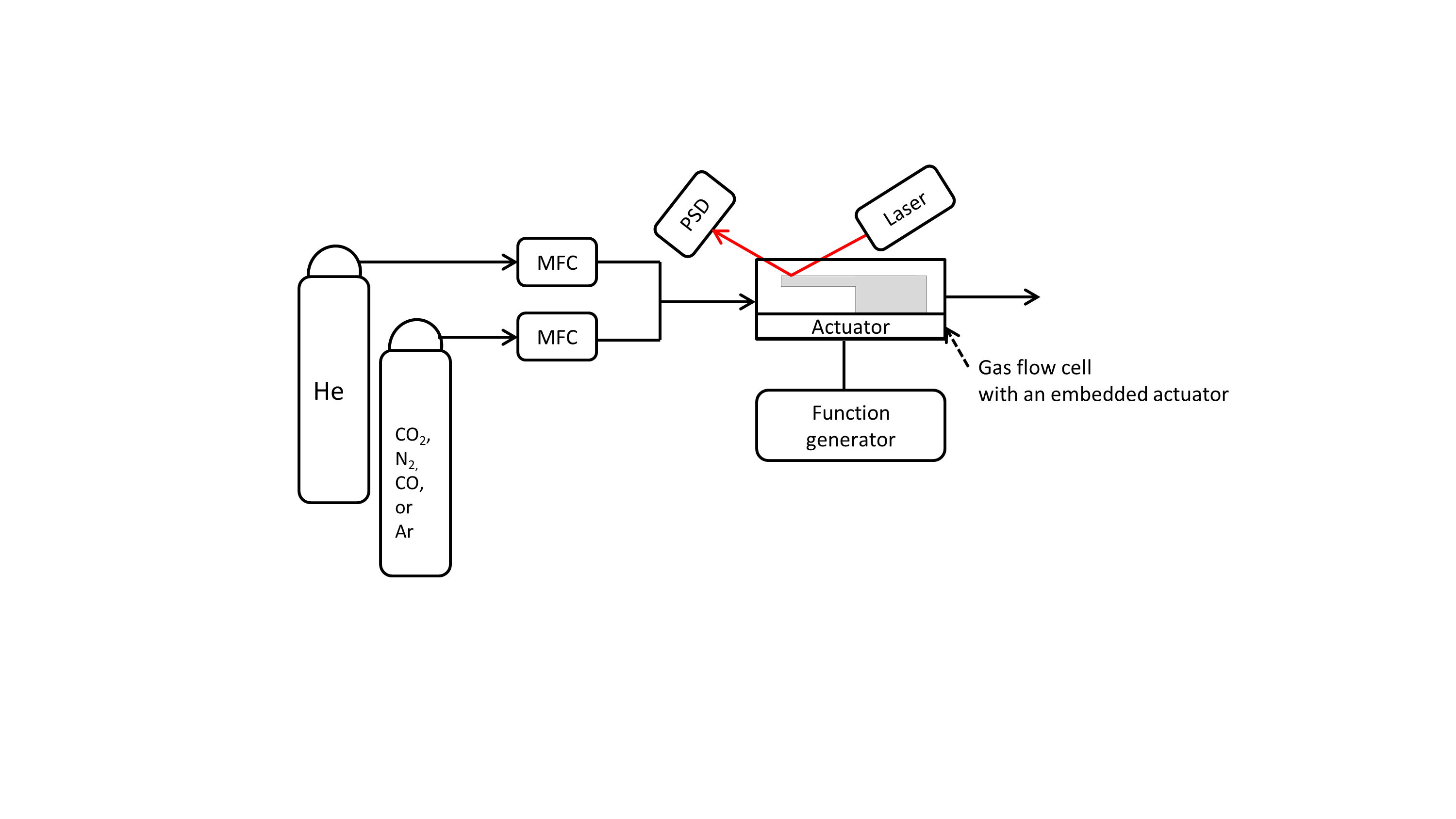
**

**Figure S5.** Schematic illustration of the instrument set-up. A focused laser beam was reflected off the gold-coated cantilevers and their deflections were recorded with a position-sensitive detector (SiTek Electro Optics, Partille, Sweden). A fast Fourier transform (FFT) algorithm was used to convert the voltage changes due to the vibrations of the cantilevers to resonance peaks from which the resonance frequencies were calculated. All the experiments were conducted at room temperature.
